# Longitudinal Analysis of Memory B and T Cell Responses to Dengue Virus in a 5-Year Prospective Cohort Study in Thailand

**DOI:** 10.3389/fimmu.2019.01359

**Published:** 2019-06-13

**Authors:** Luis A. Sánchez-Vargas, Sonia Kounlavouth, Madison L. Smith, Kathryn B. Anderson, Anon Srikiatkhachorn, Damon W. Ellison, Jeffrey R. Currier, Timothy P. Endy, Anuja Mathew, Alan L. Rothman

**Affiliations:** ^1^Institute for Immunology and Informatics, University of Rhode Island, Providence, RI, United States; ^2^Department of Medicine, University of Minnesota Medical School, Minneapolis, MN, United States; ^3^Department of Virology, Armed Forces Research Institute of Medical Sciences, Bangkok, Thailand; ^4^Viral Diseases Branch, Walter Reed Army Institute of Research, Silver Spring, MD, United States; ^5^Department of Microbiology and Immunology, State University of New York-Upstate Medical University, Syracuse, NY, United States

**Keywords:** dengue, T cells, cytokines, IFN-γ, IL-2, B cells, antibodies, ELISPOT

## Abstract

Prior exposure to dengue virus (DENV) has a profound impact on the outcome of infection, which varies according to the interval between infections. Antibodies secreted by B cells and cytokines secreted by T cells are thought to contribute both to protective immunity against DENV and the pathogenesis of dengue disease. We analyzed peripheral blood mononuclear cells (PBMC) collected from Thai children over a 5-year prospective cohort study to define the dynamics of DENV-specific memory B and T cell responses and the impact of symptomatic or subclinical DENV infections. To measure B cell responses, PBMC were stimulated with IL-2 plus R848 and culture supernatants were tested for DENV-binding antibodies by ELISA. To measure T cell responses, PBMC were stimulated in dual-color ELISPOT assays with overlapping peptide pools of structural and non-structural proteins from the four DENV types. B cell responses were low to one or more DENV types prior to symptomatic infection and increased with reactivity to all four types after infection. Subjects who had a subclinical infection or who did not experience a DENV infection during the study period showed strong memory B cell responses to all four DENV types. T cell responses to DENV peptides demonstrated a cytokine hierarchy of IFN-γ > IL-2 > IFN-γ/IL-2. T cell responses were low or absent prior to secondary infections. The trends in T cell responses to DENV peptides over 3 year post-infection were highly variable, but subjects who had experienced a secondary DENV1 infection showed higher cytokine responses compared to subjects who had experienced a secondary DENV2 or subclinical infection. The longitudinal nature of our study demonstrates persistent memory B cell responses over years and a lasting but variable impact of secondary DENV infection on DENV-specific T cell responses.

## Introduction

Immune memory is the hallmark of the adaptive immune response. The generation and persistence of memory B and T cells are key to protection against a subsequent infection with a pathogen and are the principles behind effective vaccination. Most of our understanding of durable adaptive immune responses to viruses comes from mouse models where pathogens are administered in germ-free environments under controlled conditions. Studies in mice, however, do not recapitulate the exposure in humans to multiple pathogens or even different strains of the same pathogen over time.

Dengue is the most important arthropod-borne viral disease caused by infection with any of the four dengue virus serotypes (DENV1-4) (1, 2). Most infections are subclinical but in some cases lead to clinical symptoms ranging from mild febrile illness (dengue fever, DF) to the life-threatening dengue hemorrhagic fever/dengue shock syndrome (DHF/DSS) (3). A primary infection with any of the four DENV serotypes elicits long-lasting homotypic immunity (4, 5), although there are a few reported cases of homotypic reinfections (6–9). In endemic areas, periodic inapparent exposures to DENV may boost this DENV-specific immunity (10–12). However, infection with a different DENV serotype (heterotypic secondary infection) is associated with an increased risk for DHF/DSS (4, 5, 13). Both antibodies and T cells are hypothesized to contribute to this increased risk of severe disease through a variety of mechanisms, although other data point to their ability to protect against severe DENV disease (14–20). Furthermore, evidence for a lower risk of severe disease during third or fourth DENV infections suggests that the cumulative effects of cross-reactive memory immune responses are beneficial (21–23).

The degree to which B and T cell immune responses remain stable or fluctuate over time after DENV infection may influence the balance between protective and detrimental effects of DENV-specific immune responses, but remains relatively poorly characterized. In a prospective cohort study conducted in Kamphaeng Phet province in north-central Thailand, we collected peripheral blood mononuclear cells (PBMC) over a 5-year period from children who were followed for the occurrence of DENV infections. These samples provided the opportunity to analyze the dynamics, magnitude, and durability of memory B and T cell responses to DENV over an extended period. We found that both B and T cell responses were low before symptomatic infection and increased after infection, albeit with different kinetics. In contrast, PBMC from subjects who did not experience a DENV infection during the 5-year study period had higher antibody titers at baseline and maintained broadly reactive memory B cell responses over the 5-year period. Our data demonstrate persistent memory B cell responses over years and a lasting but variable impact of secondary DENV infection on DENV-specific T cell responses.

## Materials and Methods

### Study Subjects

Subjects were selected from a 5-year prospective cohort study in Thailand that has been described elsewhere (24). In brief, blood samples from schoolchildren were collected during January of every year from 1998 to 2002. PBMC were cryopreserved in liquid nitrogen until used. In addition, sera were collected in June, August, and November of each year. Surveillance for school absences and febrile illnesses was conducted between June and November each year; acute and convalescent blood samples were collected from children with fever or history of fever. Symptomatic DENV infection was identified by febrile illness with laboratory confirmation of acute DENV infection defined by virus isolation and/or reverse transcriptase polymerase chain reaction (RT-PCR) in acute serum samples or seroconversion between acute and convalescent serum samples by IgM/IgG ELISA or hemagglutination inhibition (HAI) assay. Subclinical infection was defined by the absence of symptoms and ≥4-fold increase in HAI antibody titer from June–August or August–November. Acute primary or secondary DENV infection were distinguished by IgM/IgG ratio and HAI titers as previously described (24, 25). Subjects who consistently had no detectable antibody or stable (<4-fold change) antibody titer by HAI assay over the 5-year period were defined as having had no DENV infection during the study. Written informed consent was obtained from each subject or his/her parent or guardian. The study protocol was approved by the Institutional Review Board of the Thailand Ministry of Public Health, the Human Use Review and Regulatory Agency of the Office of the U.S Army Surgeon General and the Institutional Review Board of the University of Massachusetts School of Medicine.

### Detection of Memory B Cell-Derived Total IgG, DENV-Specific IgG, and DENV-Specific IgG Subclasses

Approximately 1–2 ×10^6^ PBMC were stimulated with 2.5 μg/ml of R848 (Invitrogen, San Diego, CA, USA) and 1,000 U/ml of IL-2 (Peprotech, Rocky Hill, NJ, USA) in a 48 well plate for 7 days at 37°C and 5% CO_2_. Culture supernatants were tested for total IgG by Human IgG ELISA Quantitation Set according to the manufacturer's instructions (No. E80-104, Bethyl Laboratories, Inc., Montgomery, TX, USA). Total IgG levels in the supernatants from stimulated PBMC ranged from 4.21 to 32.73 μg/ml (mean 16.92 μg/ml). Samples with a concentration below the limit of detection (1.56 μg/ml) were excluded. DENV-specific IgG and the four IgG subclasses (IgG1-4) were detected in the supernatant by ELISA. Briefly, 96 well plates were coated overnight with 10 ng/well of DENV1-4 virus-like particles (VLPs) (Native Antigen Company). The plates were blocked for 90 min with 1% BSA. The diluted culture supernatant was added to the wells for 60 min. Plates were washed and goat anti-human IgG coupled to HRP (A80-104P; Bethyl Laboratories Inc., Montgomery, TX, USA) was added for total DENV-specific IgG detection. HRP conjugated secondary antibodies for IgG subclasses detection include goat anti-human IgG1 FC (9054-05), IgG1 hinge (9052-05), IgG2 FC (9060-05), IgG3 hinge (9210-05), and IgG4 FC (9200-05); all were from Southern Biotechnology Associates, Birmingham, AL, USA. The assay was developed with TMB substrate (34021; Thermo Scientific, MA, USA), stopped with 1M HCL, and read at 450 nm.

### Peptide Pools

To evaluate memory T cell responses, we used four peptide pools spanning the prM and E proteins from DENV1-4, four peptide pools spanning NS1, NS3, and NS5 proteins (NSA pools) from DENV1-4, and one peptide pool spanning NS2a/b, NS4a/b, and C protein from DENV2 (NSB pool). Peptide pools contained peptides that ranged in length from 12 to 20 amino acids (aa), in overlap from 10 to 14 aa. The total number of peptides per pool ranged from 88 to 323 aa (Table S1). Peptides were obtained from NIH Biodefense & Emerging Infections Research Resources Repository (BEI Resources, Bethesda, MD, USA) and Peptide technologies (JPT, Acton, MA, USA). Further details of the peptides are available at http://www.beiresources.org/.

### *Ex vivo* Dual Color IL-2 and IFN-γ Enzymatic ELISPOT Assay

The ELISPOT assay was performed according to the manufacturer's instructions (CTL, Cleveland, OH, USA). Cryopreserved PBMC were thawed and plated at a density of 1–2 × 10^5^ cells/well. Peptide pools were added at a final concentration of 2 μg/ml/peptide. As a positive control, PBMC were incubated with anti-CD3 and anti-CD28 antibodies at final concentrations of 1 and 0.1 μg/ml, respectively. As a negative control, PBMC were incubated with medium. PBMC were stimulated for 45 h at 37°C with 5% CO_2_. As a positive control, every plate had PBMC from a well-characterized DENV-immune subject tested with the same conditions. The number of spots per well was determined using an automated ELISPOT reader (S5UV analyzer, CTL, Cleveland, OH, USA) with the double color software. Determinations from duplicate wells were averaged. Data were analyzed by subtracting the mean number of spots in the wells with cells and medium-only from the mean counts of spots in wells with cells and antigen and expressed as spot-forming cells (SFC) per 10^6^ PBMC. If the response to anti-CD3/CD28 antibodies was below 500 IFN-γ SFC per million PBMC, the sample was excluded.

### Statistical Analysis

Statistical analysis was performed using GraphPad Prism software V 8.00 (GraphPad Software Inc., La Jolla, CA). The non-parametric Mann-Whitney *U* or Wilcoxon signed rank test was used to compare two groups as appropriate. Statistical significance was set at *P* < 0.05.

## Results

### Characteristics of the Study Population

PBMC from 27 subjects were selected for study based on available data from 5-years of follow-up (Table S2). Sixteen subjects were selected for B cell assays: six who had symptomatic DENV infection, four who had a subclinical infection and six subjects with no DENV infection. Twenty-five subjects were selected for T cell assays: 13 who had a symptomatic secondary DENV infection (six with DENV1, six with DENV2, and one without a serotype determined), six who had a subclinical infection, and six with no DENV infection. For the symptomatic and subclinical infection groups, we selected subjects who experienced an infection in the first or second year of the study to allow us to test PBMC collected 1–2 years before and 3–4 years after secondary DENV infection. HAI antibody titers to the four DENV serotypes at baseline (start of the study) were higher in the subclinical and no DENV infection groups compared to the symptomatic DENV1 and DENV2 groups (*p* < 0.05) (Figure S1).

### Memory B Cell Responses to DENV

**Figure 1:**
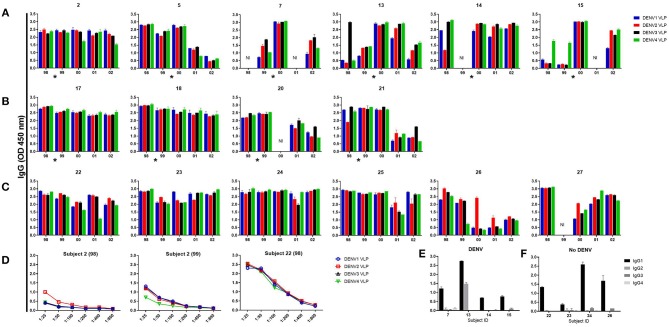
Memory B cell responses to DENV. Peripheral blood mononuclear cells from 6 subjects with symptomatic secondary DENV infection (subjects 2 and 5 were not hospitalized, subjects 7, 13, 14, and 15 were hospitalized) **(A)**, 4 subjects with subclinical infection **(B)**, and 6 subjects with no DENV infection **(C)** were cultured with R848 plus IL-2 for 7 days and supernatants were tested for DENV-specific IgG Abs. Representative data of supernatant dilution from a subject with symptomatic and no DENV infection **(D)**. DENV-specific IgG isotypes in supernatants from the year 2000 in 4 subjects with DENV and 4 subjects with no DENV, respectively **(E,F)**. Bars represent mean with standard error of the mean of the OD values. The asterisk indicates the approximate time when DENV infection occurred. NI, not included.

To study the dynamics, magnitude, and durability of the memory B cell (MBC) responses to DENV, PBMC from 16 subjects were stimulated with IL-2 plus R848. Dengue-specific IgG antibodies were assessed in supernatants of stimulated PBMC by ELISA using VLPs from each of the four DENV serotypes (Figures 1A–C). A total of 74 PBMC samples were analyzed. DENV-specific MBC responses were low to the infecting serotype prior to secondary DENV infection in hospitalized subjects 7, 13, 14, and 15 while they were high to all four serotypes in two subjects who were not hospitalized (Figure 1A, Figure S2A). For some subjects, MBC responses were low to one or more additional DENV serotypes. MBC responses increased in the first year after infection, with cross-reactivity to all four serotypes suggesting an expansion of DENV-cross-reactive B cells (Figure 1A, Figure S2). The increased MBC response was maintained in PBMC from two of four hospitalized individuals with symptomatic infection (subjects 13 and 14) for at least 3–4 years after infection (Figure 1A). In contrast, MBC responses to all four DENV serotypes were high and persisted over the 5 years in 2 of 4 subjects with subclinical infection and 5 of 6 subjects studied who did not experience a DENV infection over the 5-year period of observation (Figures 1B,C). Serial dilution of supernatants did not reveal preferential binding to a specific DENV serotype (Figure 1D, Figure S2). DENV-specific antibodies detected in MBC culture supernatants were predominantly of the IgG1 subclass (Figures 1E,F). IgG subclass-specific responses did not differ between subjects with symptomatic vs. no DENV infection.

### Memory T Cell Responses to DENV

To assess the dynamics, magnitude, and durability of the memory T cell responses to DENV, 106 PBMC samples from 25 subjects were stimulated with peptide pools of structural and non-structural proteins. DENV-specific memory T cells producing IFN-γ, IL-2, or IFN-γ/IL-2 were determined by dual color ELISPOT. Because of the limited number of cells available, PBMC from subjects who experienced a symptomatic DENV1 or DENV2 infection were stimulated with DENV1 or DENV2 prM/E peptides, respectively, in addition to all non-structural peptide pools. PBMC from subjects with a subclinical or no DENV infection were stimulated with prM/E peptides from all four DENV serotypes in addition to the non-structural peptide pools (Table S1).

**Figure 2:**
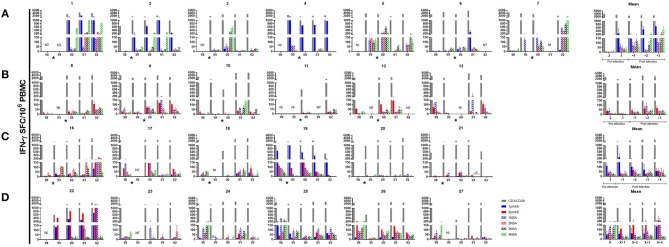
IFN-γ ELISPOT responses in individual subjects. DENV peptide-specific interferon γ (IFN-γ) spot-forming cells (SFC) were determined by dual color ELISPOT in PBMC collected at the indicated time points. PBMC were collected at the beginning of each year. Subjects who experienced a **(A)** DENV1 infection **(B)** DENV2 infection **(C)** subclinical dengue infection and **(D)** no dengue infection over the 5-year period. Bars represent mean with standard error of the mean for duplicate wells. The asterisk indicates the approximate time when DENV infection occurred. NT, not tested; NI, not included.

Overall, T cell responses to DENV peptide pools demonstrated a hierarchy of cytokine production of IFN-γ > IL-2 > IFN-γ/IL-2 in all subjects (Figure S3). DENV-specific IFN-γ responses were low or absent prior to symptomatic DENV infection. Responses increased after infection in most of the subjects (Figure 2, Figure S4). However, the peak frequency of DENV-specific IFN-γ-producing memory T cells was not uniformly detected in the first sample collected after infection. Overall, the difference in magnitude of DENV-specific IFN-γ responses before vs. after infection did not reach statistical significance. DENV-specific T cells were somewhat more stable over the 5-year period in subjects who did not experience a DENV infection (Figure 2).

DENV-specific IFN-γ responses in PBMC collected in the first year of the study (baseline responses) reflect immune responses potentially predictive of outcome. There were no significant differences in baseline DENV-specific IFN-γ responses between subjects who subsequently had a symptomatic infection vs. those who had a subclinical infection. However, baseline responses tended to be higher in subjects who did not experience a DENV infection compared to the other groups (symptomatic or subclinical DENV infection; Figure S5).

DENV-specific IFN-γ responses in PBMC collected in later years of the study assess the effects of secondary DENV infection on memory T cell responses. There was a trend toward higher IFN-γ responses following symptomatic DENV1 infection compared to the other groups (symptomatic DENV2, subclinical or no DENV infection), but this did not reach statistical significance (Figure 2, Figure S6). As expected, PBMC collected post-infection showed IFN-γ responses to more than one DENV serotype (Figure 2).

## Discussion

We conducted a longitudinal analysis of memory B and T cell responses to DENV over a 5-year period in primary schoolchildren living in a dengue-endemic area. Subjects were chosen to elucidate the impacts of incident DENV infections, both clinical and subclinical, on DENV-specific immunological memory, to follow changes in DENV-specific immunological memory over 3+ years after infection, and to compare findings in subjects who had symptomatic, subclinical, or no DENV infections during the study period. Other studies have measured memory B and T cell responses to DENV, however, these have largely been cross-sectional studies of individuals following natural DENV infection or vaccination (19, 26–33). The longitudinal nature of our study, availability of PBMC samples both before and after DENV infection, and the inclusion of groups with different DENV experience led to several novel observations.

Our assay of memory B cell responses to DENV used ELISA to evaluate binding to virions by antibodies secreted from circulating memory B cells after *in vitro* stimulation. Responses were detected in PBMC collected prior to an incident DENV infection. This finding is consistent with the known high baseline seroprevalence in this population and supports the secondary nature of most infections in our cohort even when titers of HAI and neutralizing antibody were undetectable (15, 34, 35). These samples also showed lower responses to one or more serotype(s), which in each case included the serotype that caused the next infection. This finding suggests that assays of memory B cells might help to assess susceptibility to infection among DENV-experienced individuals, which has been difficult to define based on serum assays (14, 36). The high responses to all four serotypes in study subjects who did not experience a DENV infection may therefore reflect protective immunity in those subjects (37).

Following an incident DENV infection, memory B cell responses increased in magnitude and breadth, consistent with a model wherein serotype-cross-reactive memory B cells are preferentially expanded by sequential DENV infections (32, 33, 38). However, while memory B cell responses increased in the earliest blood sample collected after infection, there was substantial heterogeneity in the durability of this increase between study subjects and, for each subject, across viral serotypes. Human memory B cells have been detected decades after infection or vaccination, but are less durable for some vaccines and pathogens (39–44). Our data imply an important role of subject-specific and cell-intrinsic factors in the durability of DENV-specific memory B cells (44). Further studies are needed to define these factors and their relationship to susceptibility or resistance to subsequent DENV infection or disease.

We measured memory T cell responses to DENV using dual IFN-γ/IL-2 ELISPOT assays and stimulation with overlapping peptide pools. In contrast to memory B cell responses, we did not detect memory T cell responses in all subjects. Whereas, some studies of adult volunteers after natural DENV infection or administration of live virus vaccine candidates have reported DENV-specific memory T cell responses in nearly all subjects (26, 45, 46), our results are in line with other cross-sectional studies of dengue-endemic populations (15, 16, 19, 27, 29, 47–50). Other factors that may contribute to lower memory T cell responses in our study include the young age of our subjects, the number of prior exposures to DENV, and other endemic infections (51, 52). Memory T cell responses were generally lower in those subjects who experienced a symptomatic DENV infection, as we observed for memory B cells. There have been very few prospective studies of DENV-specific memory T cell responses in PBMC collected prior to secondary DENV infection (15, 49, 53). An inverse relationship was previously found in our cohort between DENV-specific T cell responses measured by intracellular staining for IFN-γ or IL-2 and symptomatic DENV infection (15). Others reported an inverse correlation between DENV-specific IFN-γ ELISPOT responses and HLA class I-related susceptibility to dengue (16).

Memory T cell responses to DENV were higher in the PBMC collected after an incident DENV infection, but these responses were quite variable over a 3-year period, and responses were not consistently highest in the samples collected closest in time to the infection. Previous studies have either been cross-sectional in nature (26, 27, 29, 30) or covered a short time period (46, 47, 54), and therefore are not directly comparable to our findings. It is possible that measurement of other T cell functions would have reflected different trends in cell frequency (55–57). Given ongoing DENV transmission in the study area throughout the period of observation, it is also possible that some subjects experienced additional exposures to DENV that modulated T cell frequency but were not detected as significant increases in antibody titer.

Our findings must be interpreted in the context of several limitations of our study design. The quantity of PBMC available from the small blood volumes collected from young children was a major consideration for assay design. As a result, we could not test all antigen specificities or effector functions of memory B and T lymphocytes. Antibody responses to NS1 (58), T cell responses to other structural and non-structural antigens (59, 60), and multiparameter single-cell analyses (46, 61) would be of interest and might modify some of our conclusions. We used pools containing a large number of overlapping peptides in T cell ELISPOT assays, which has been reported to be suboptimal for detection of responses (19, 46, 62–64). We studied memory T and B cell responses in PBMC; lymphocyte repertoires in blood and tissue have been shown to differ in some infections, such as influenza (65). As DENV causes systemic infection, the most relevant tissues have not been defined, and most other studies of the immune repertoire have utilized PBMC. The narrow criteria we used for selection of cohort subjects limited the number available for these studies and reduced the statistical power for comparisons between groups. Our cohort study design along with these criteria also restricted the age range, host genetics, and geographic distribution of study subjects, which is likely to have limited the diversity of prior DENV infection history (66). Comparable studies in other populations will be needed to determine the generalizability of our findings.

Overall, this longitudinal analysis of memory B and T cell responses to DENV and the impacts of intervening symptomatic and subclinical DENV infections supports the paradigm of selective expansion of DENV-specific immunological memory by sequential exposures, but reveals additional heterogeneity between and within individual subjects than could be ascertained from cross-sectional studies. This heterogeneity should be considered in the interpretation of data from cohort studies of natural DENV infection and clinical trials of dengue vaccines. We suggest that further studies are warranted to evaluate the associations with susceptibility vs. resistance to DENV infection and disease.

## Ethics Statement

Written informed consent was obtained from each subject or his/her parent or guardian. The study protocol was approved by the Institutional Review Board of the Thailand Ministry of Public Health, the Human Use Review and Regulatory Agency of the Office of the U.S Army Surgeon General and the Institutional Review Board of the University of Massachusetts School of Medicine.

## Author Contributions

LS-V, AS, AM, and AR conceived and designed the experiments and wrote the manuscript text. LS-V, SK, and MS performed experiments and prepared figures. LS-V conducted statistical analyses. AS, KA, DE, and TE supervised the clinical study, subject enrollment, and collection of clinical data and blood samples. AS, KA, and DE contributed to the analysis of clinical, virologic, and serologic data. JC contributed to the analysis of immunological data. All authors contributed to the final manuscript and agree with the results and conclusions.

### Conflict of Interest Statement

AR has received compensation as a consultant to Sanofi Pasteur, Takeda, and Merck. The remaining authors declare that the research was conducted in the absence of any commercial or financial relationships that could be construed as a potential conflict of interest.
