# Supplementary material for: Longitudinal Analysis of Memory B and T Cell Responses to Dengue Virus in a 5-Year Prospective Cohort Study in Thailand
Source: Front Immunol. 2019 Jun 13;10:1359. doi: 10.3389/fimmu.2019.01359 (PMC6585174; doi:10.3389/fimmu.2019.01359)
Supplement: Supplementary file 1 [file Presentation_1.pdf]

Table S1. Peptide arrays for the T cell study

Peptide pool	Dengue strain	Peptides per protein	Length	Overlaps	Total number of peptides
1prM/E	Nauru/WestPac/1974	prM; 34 peptides	16-mers	11aa	118
	Singapore/S275/1990	E; 84 peptides	13- to 18-mers	11 or 12 aa	
2prM/E	New Guinea C (NGC)	prM; 21 peptides	15- to 20-mers	10 aa	88
		E; 67 peptides		10 or 11 aa	
3prM/E	CH53489	prM; 34 peptides	16-mers	11aa	102
	Sleman/1978	E; 68 peptides	12- to 20-mers	10 or 11 aa	
4prM/E	Singapore/8976/1995	prM; 34 peptides	16-mers	11aa	103
	Dominica/814669/1981	E; 69 peptides	12- to 20-mers	10 or 11 aa	
1NS group A	Singapore/S275/1990	NS1; 61 peptides	13- to 17-mers	11 or 12 aa	322
		NS3; 106 peptides	14- to 17-mers		
		NS5; 155 peptides	12- to 17-mers		
2NS group A	New Guinea C (NGC)	NS1; 47 peptides	15- to 19-mers	10 or 11 aa	286
		NS3; 83 peptides	13- to 19-mers	10 aa	
		NS5; 156 peptides	15- to 17-mers	11 to 13 aa	
2NS group B	New Guinea C (NGC)	C; 14 peptides	15- to 18-mers	10 aa	137
		NS2a; 35 peptides	15- to 17-mers	11 aa	
		NS2b; 22 peptides	13- to 17-mers	11 to 14 aa	
		NS4a; 48 peptides	14- to 17-mers	11 or 12 aa	
		NS4b; 18 peptides	12- to 17-mers	11 aa	
3NS group A	Philippines/H87/1956	NS1; 60 peptides	13- to 17-mers	11 or 12 aa	321
		NS3; 105 peptides	14- to 17-mers	11 or 12 aa	
		NS5; 156 peptides	13- to 17-mers	11 to 13 aa	
4NS group A	Singapore/8976/1995	NS1; 61 peptides	13- to 17-mers	11 or 12 aa	323
		NS3; 106 peptides	15- to 17-mers	11 or 12 aa	
		NS5; 156 peptides	13- to 17-mers	11 to 14 aa	

Table S2. Characteristics of the study population

Subject no.	Year of infection	Serology	Serotype	Clinical manifestation	Study	Interval pre (mo)[a]	Interval post (mo)[b]
1	1998	Secondary	DENV1	NH	T cells	4	7
2	1998	Secondary	DENV1	NH	T & B cells	5	7
3	1998	Secondary	DENV1	HDF	T cells	5	7
4	1999	Secondary	DENV1	NH	T cells	5	7
5	1999	Secondary	DENV1	NH	T & B cells	4	8
6	1999	Secondary	DENV1	HDHF	T cells	5	8
7	1999	Secondary	ND	HDHF	T & B cells	4	8
8	1999	Secondary	DENV2	NH	T cells	5	7
9	1999	Secondary	DENV2	NH	T cells	5	8
10	1999	Secondary	DENV2	NH	T cells	4	9
11	1999	Secondary	DENV2	HDHF	T cells	6	6
12	1999	Secondary	DENV2	NH	T cells	6	7
13	1999	Secondary	DENV2	HDF	T & B cells	6	6
14	1999	Secondary	DENV2	HDF	B cells	6	6
15	1999	Secondary	ND	HDHF	B cells	5	7
16	1998	Secondary	NT	Subclinical	T cells	6-9	2-5
17	1998	Secondary	NT	Subclinical	T & B cells	0-3	9-12
18	1998	Secondary	NT	Subclinical	T & B cells	3-6	6-9
19	1998	Secondary	NT	Subclinical	T cells	6-9	2-6
20	1998	Secondary	NT	Subclinical	T & B cells	0-4	8-12
21	1998	Secondary	NT	Subclinical	T & B cells	0-4	8-12
22	NA	Immune	NT	No dengue	T & B cells	NA	NA
23	NA	Immune	NT	No dengue	T & B cells	NA	NA
24	NA	Immune	NT	No dengue	T & B cells	NA	NA
25	NA	Immune	NT	No dengue	T & B cells	NA	NA
26	NA	Immune	NT	No dengue	T & B cells	NA	NA
27	NA	Immune	NT	No dengue	T & B cells	NA	NA

NH, nonhospitalized; HDF, hospitalized dengue fever; HDHF, hospitalized dengue hemorrhagic fever; NA, not applicable; ND, not determined; NT, not tested; [a] interval from last pre-infection blood sample to infection (estimated for subclinical infections); [b] interval from infection to first post-infection blood sample (estimated for subclinical infections)

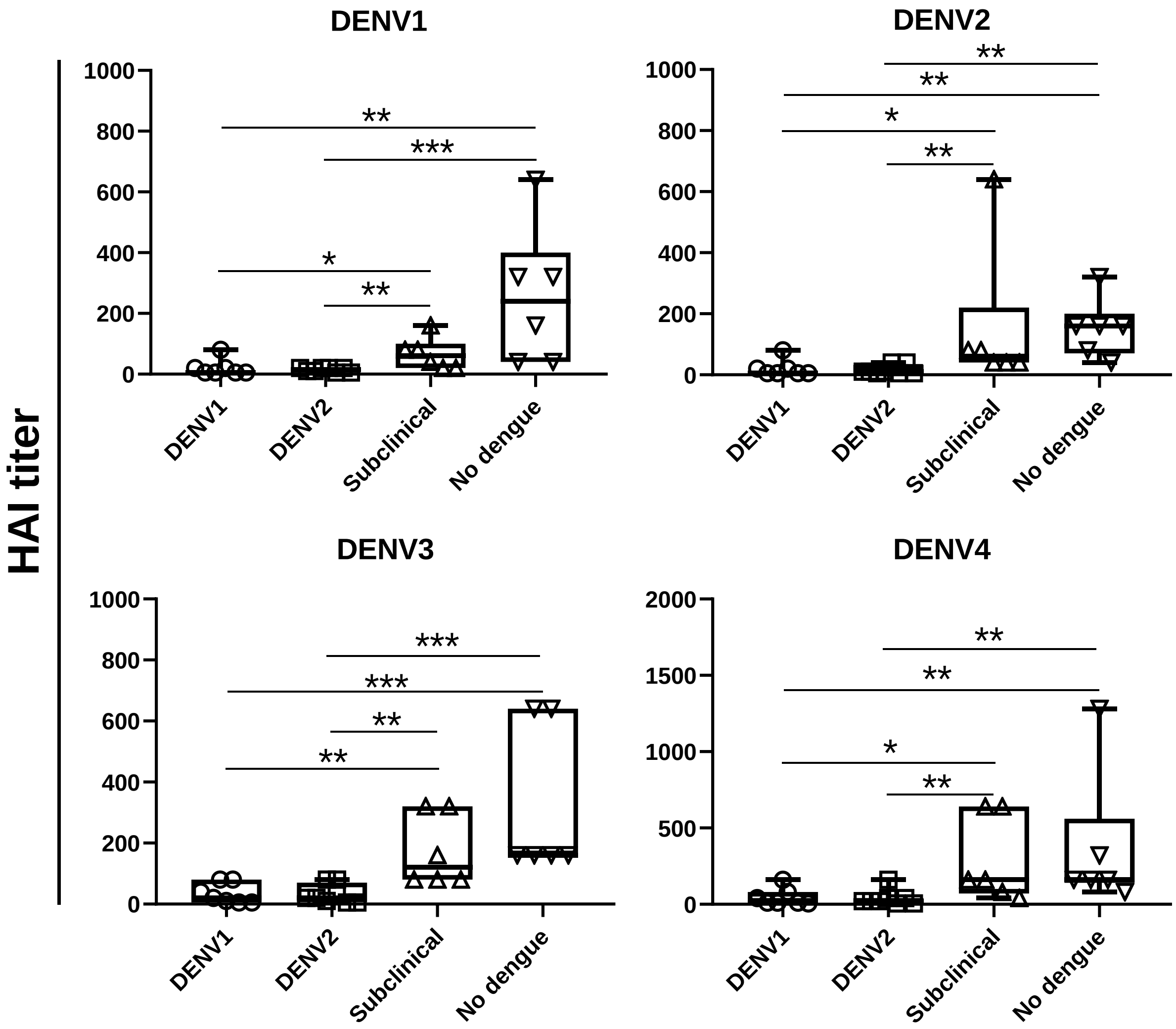

Figure S1. Baseline HAI titers to the four dengue virus serotypes in each group. HAI titers to each serotype at baseline were compared between groups. Symbols represent the HAI titer on each subject. The box and whiskers represent 25% to 75% percentile, and min and max values, respectively. Statistics were calculated by the non-parametric Mann-Whitney U test. * $p < 0.05$, ** $p < 0.01$, *** $p < 0.001$

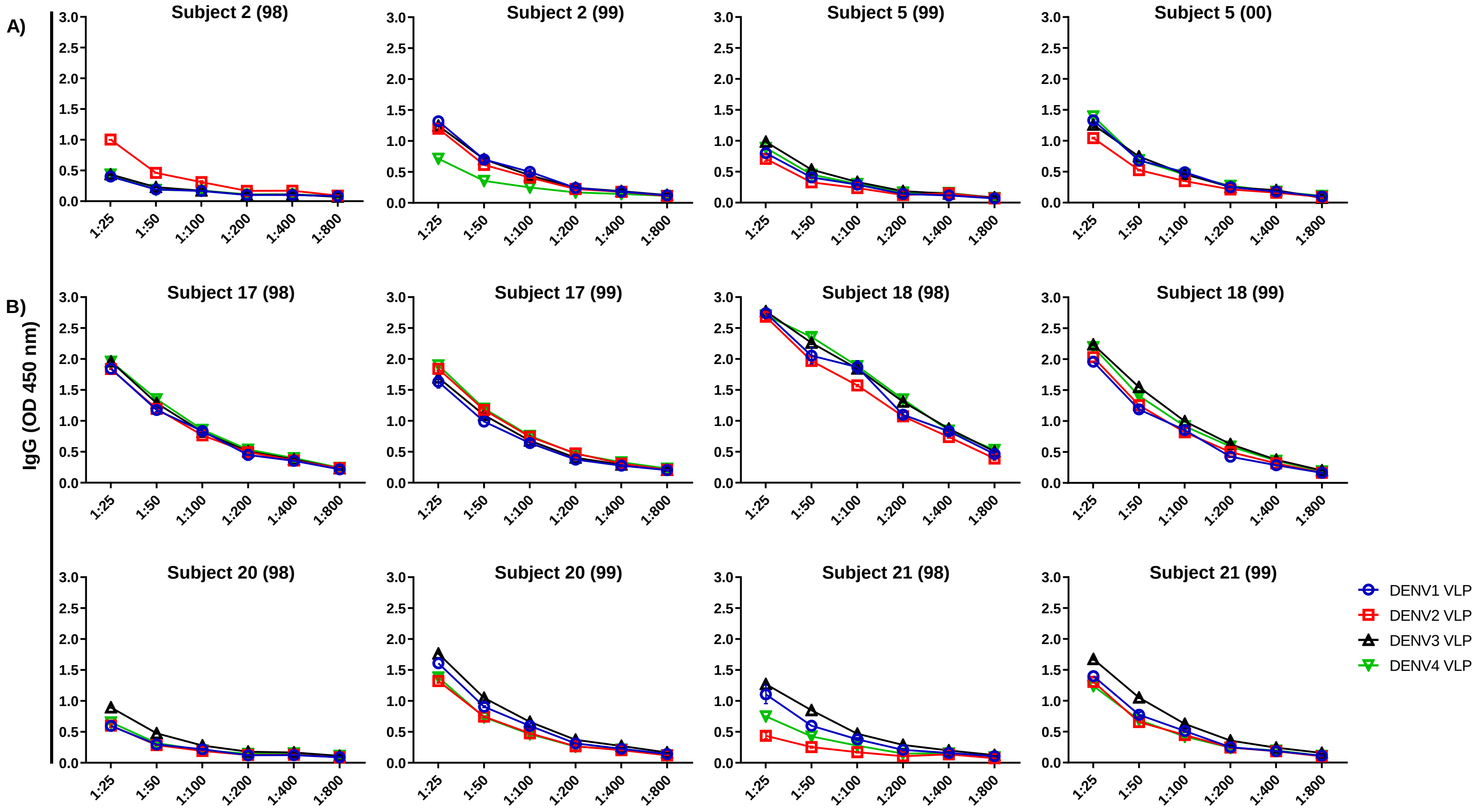

Figure S2. DENV-specific IgG titration of memory B cell-derived supernatants. Serial dilutions of culture supernatants before and after secondary dengue infection from two subjects with symptomatic A) and four subjects with subclinical infection B) were analyzed.

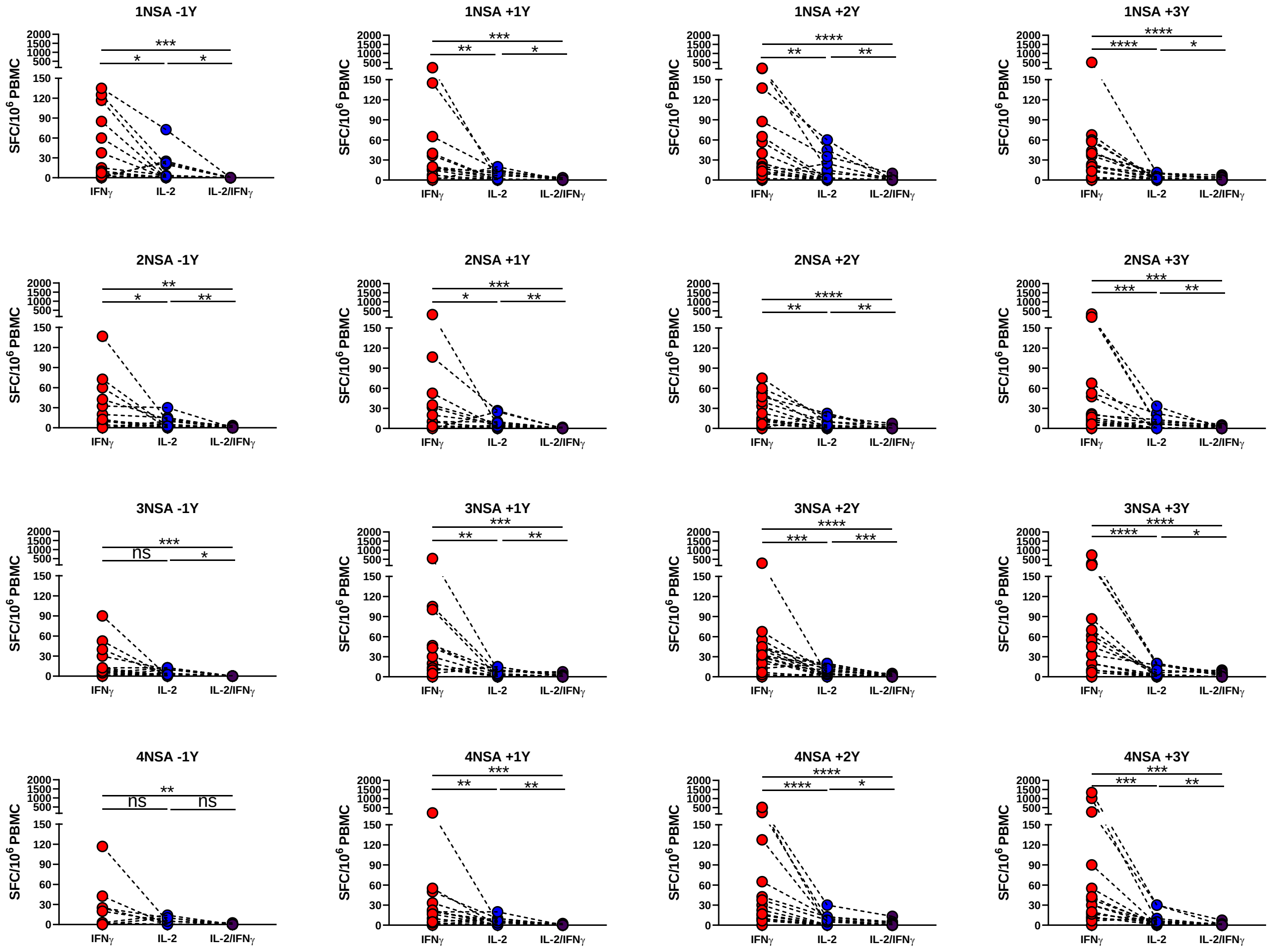

Figure S3. Dominant production of IFN-γ over IL-2 or IFN-γ+ IL-2+ cells by DENV-specific T cells. Serotype-specific IFN-γ, IL-2 and IL-2/IFN-γ spot forming cells (SFC) were detected by dual color ELISPOT in response to NS group A peptides at 1 year prior (-1Y), 1 (+1Y), 2 (+2Y) or 3 (+3Y) years post infection in PBMC from symptomatic and subclinical dengue subjects. Circles represent the frequency of cytokine producing cells in PBMC from each subject. Statistics were calculated by the non-parametric Wilcoxon matched-pairs signed rank test. * $p < 0.05$, ** $p < 0.01$, *** $p < 0.001$, **** $p < 0.0001$

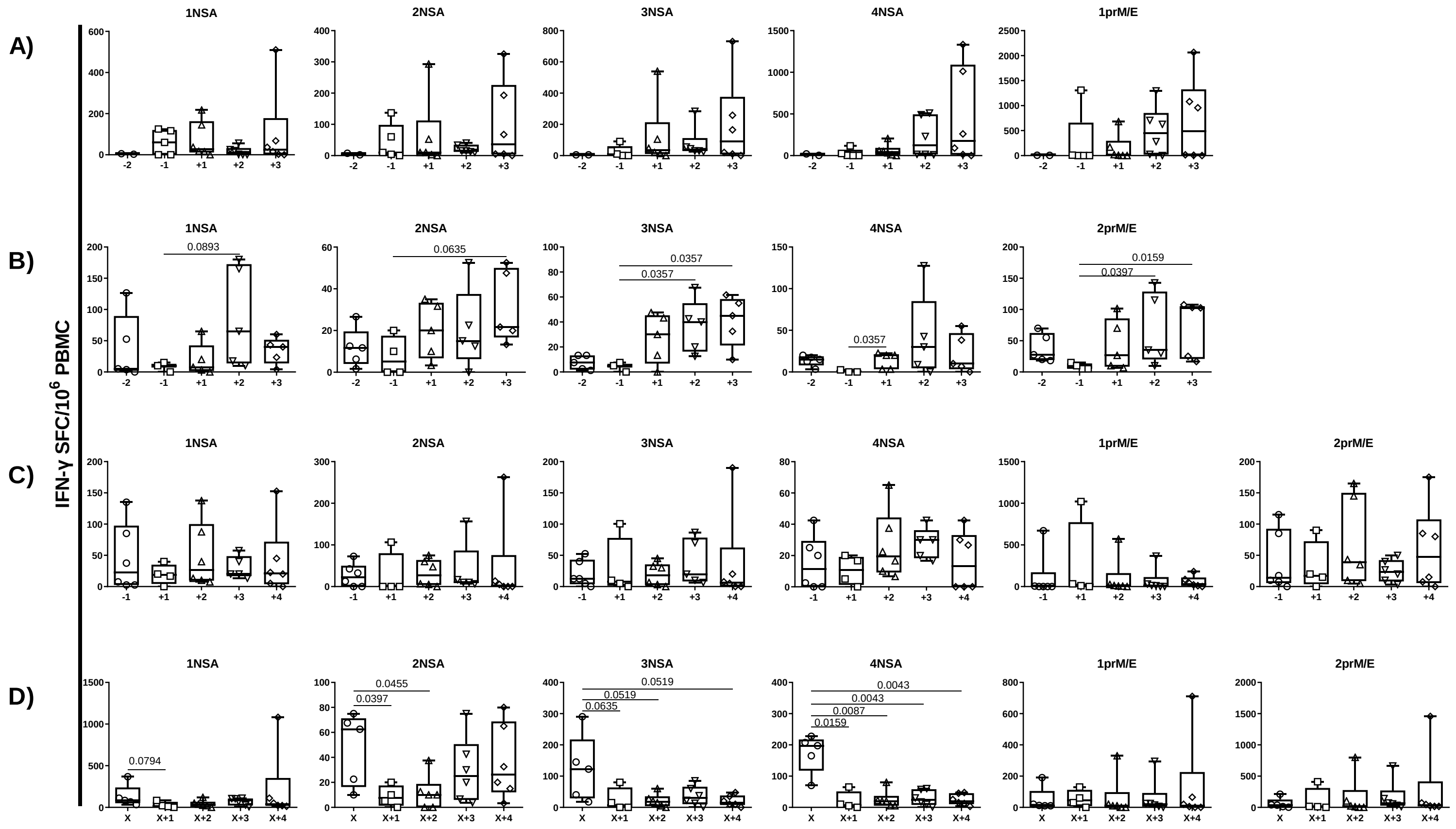

Figure S4. Trends in DENV peptide-specific IFN-γ producing T cell frequencies over the 5-year study period. Frequencies of IFN-producing T cells were compared at the indicated time points among subjects with DENV1 infection (A), DENV2 infection (B), subclinical DENV infection (C), and no DENV infection (D). The x axis indicates time points before (-1) or after (+1, +2, +3, +4) the incident infection; for group D, x indicates the baseline sample and subsequent samples are at yearly intervals (x+1, etc). Symbols represent the frequencies of IFN-producing T cells from each subject. The box and whiskers represent 25% to 75% percentile, and min and max values, respectively. Statistics were calculated by the non-parametric Mann-Whitney U test.

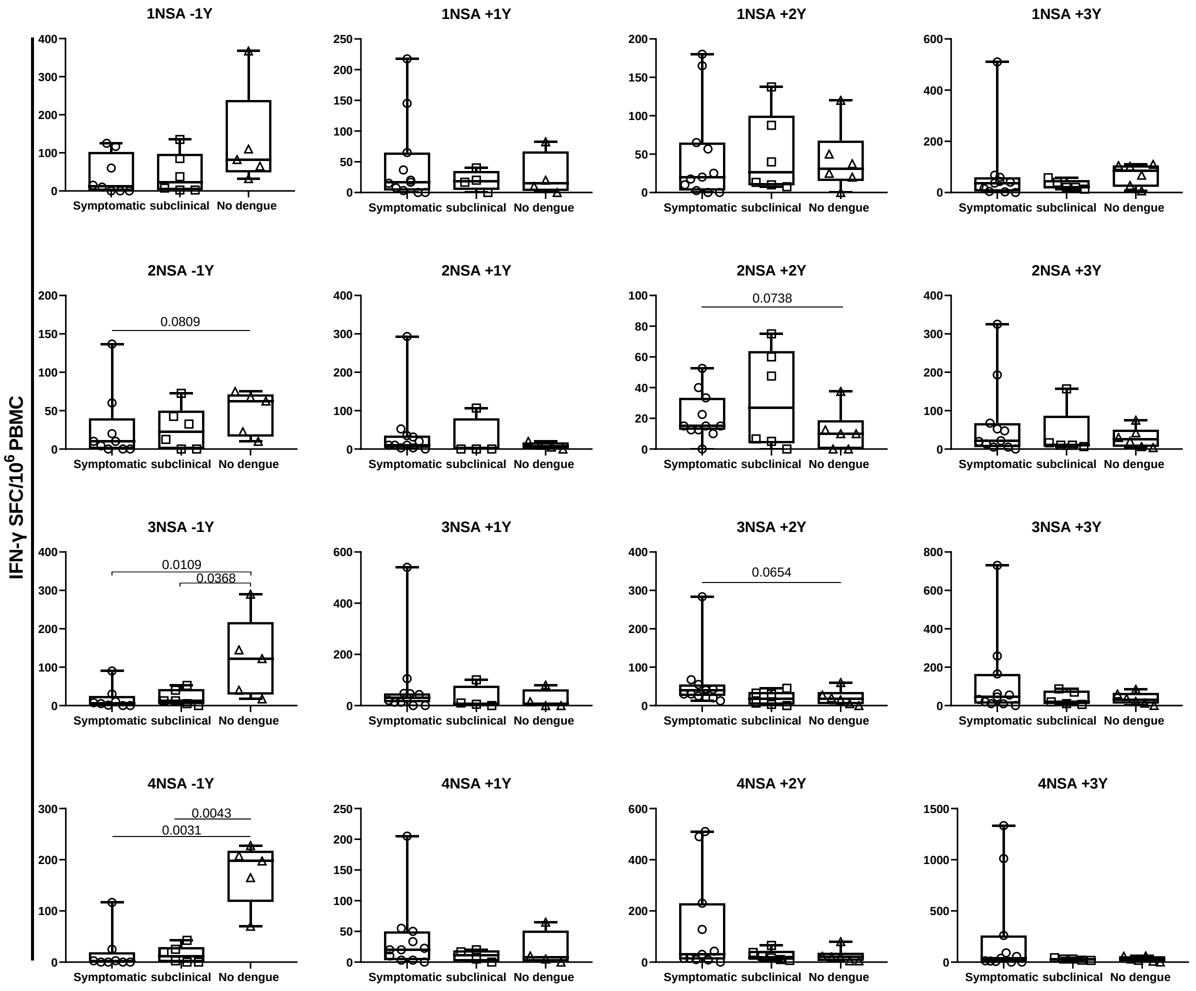

Figure S5. DENV peptide-specific T cell responses, by clinical manifestations. IFN-producing T cells were compared at the indicated time point between symptomatic, subclinical and no DENV group. Symbols represent the frequencies of IFN-producing T cells from each subject. The box and whiskers represent 25% to 75% percentile, and min and max values, respectively. Statistics were calculated by the non-parametric Mann-Whitney U test.

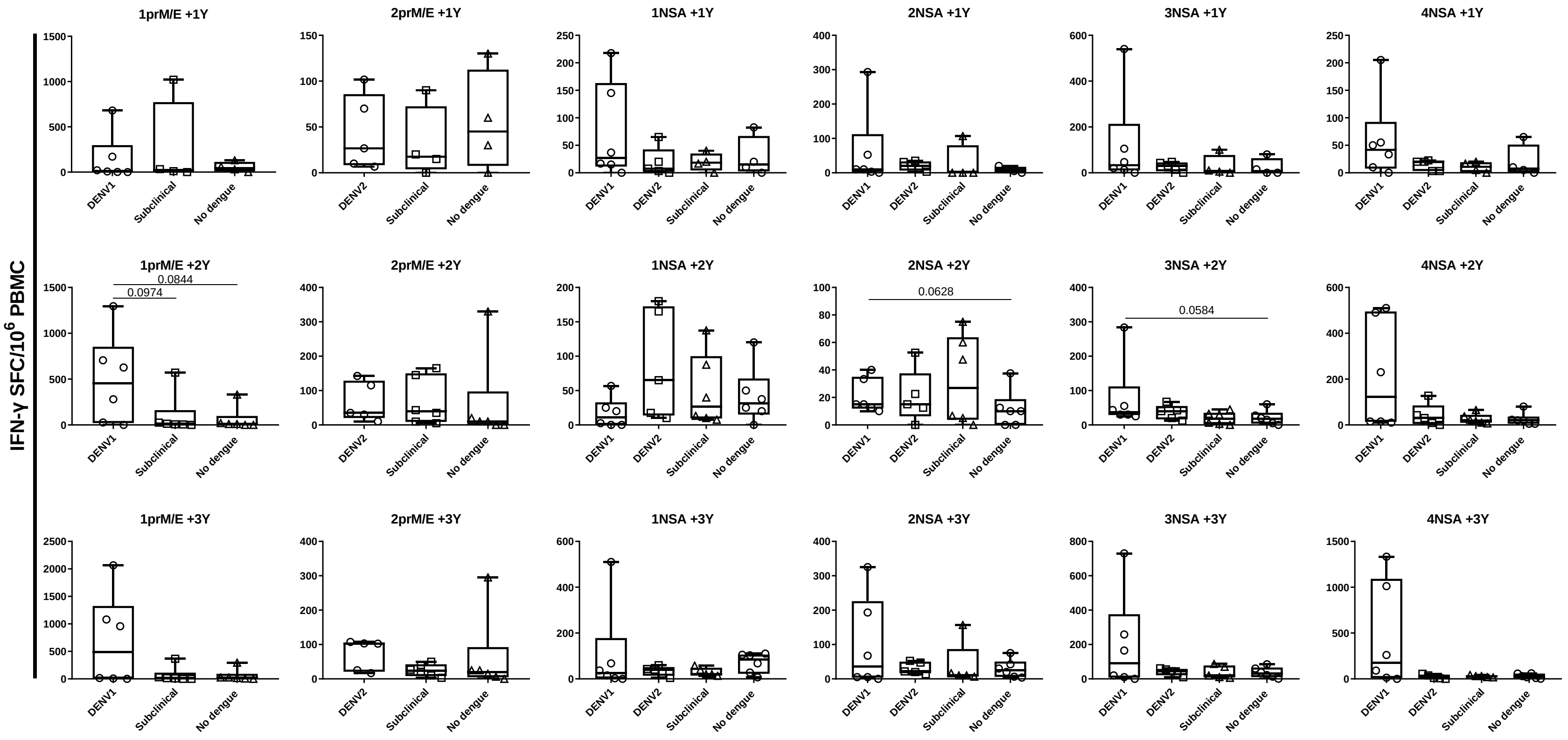

Figure S6. DENV peptide-specific IFN-γ responses after secondary infection, by serotype of infection. IFN-producing T cells were compared at the indicated time point between the groups. Symbols represent the frequencies of IFN-producing T cells from each subject. The box and whiskers represent 25% to 75% percentile, and min and max values, respectively. Statistics were calculated by the non-parametric Mann-Whitney U test.
